# Secondary school teachers' use of online formative assessment during COVID‐19 lockdown: Experiences and lessons learned

**DOI:** 10.1111/jcal.12699

**Published:** 2022-07-05

**Authors:** Maria Joanna Veugen, Judith Theresia Maria Gulikers, Perry den Brok

**Affiliations:** ^1^ Education and Learning Sciences Wageningen University and Research Wageningen The Netherlands

**Keywords:** formative assessment cycle, online formative assessment, online learning, secondary education, teacher development

## Abstract

**Introduction:**

During the COVID‐19 lockdown in 2020, teachers had to shift their teaching and assessment to online. Formative assessment (FA) can help teachers to engage, guide and monitor students' (online) learning. However, more knowledge is needed of how teachers could use the full FA process online.

**Methods:**

In this study data from 50 secondary school teachers, who taught different grade levels and subjects and joined a FA learning network that started before and continued during the lockdown, were collected. The study investigates how they used online FA practices differently than face‐to‐face FA, what challenges and opportunities they experienced in online FA and what lessons they learned and intended to keep. This mixed methods study used data from a questionnaire, interviews and webinars that were segmented, coded and analysed.

**Results:**

Results showed that many teachers implemented new FA strategies and adopted, more often than in their face‐to‐face practice, all the five phases of the FA process in an aligned matter in online FA. Teachers indicated opportunities in stimulating student engagement and guiding and monitoring student learning more at an individual level in the online FA process, but also experienced challenges, mainly in lack of interaction online.

**Discussion:**

The sudden and necessary shift to online FA, due to the COVID‐19 lockdown, challenged teachers to more fundamentally reconsider their assessment practices and assumptions. Teachers intended to make use of these learned lessons to improve their future (blended) FA practice.

## 1 INTRODUCTION

Since the COVID‐19 lockdown periods in 2020, online education has made a tremendous transformation. Schools were physically closed worldwide for certain periods and students were taught by their teachers online in virtual classrooms (The Organization for Economic Cooperation and Development [OECD], 2020; United Nations Educational, Scientific and Cultural Organization [UNESCO], 2020). Secondary school teachers had to suddenly shift their teaching practice to online, which resulted in adapted lesson plans and alternative ways to monitor students' learning processes (König et al., 2020; OECD, 2020). This was also the case in the Netherlands, in which all education had to shift suddenly to online and all teachers had to adapt their digital skills, even the ones who were not used in doing so before, to educate their students via Zoom or other platforms (Van der Spoel et al., 2020). Teachers had to design this online education within a week and were not guided in how they should do so at the start of the lockdown. This sudden shift to online teaching came with its challenges for teachers, especially in engaging all students and guiding and monitoring student learning (König et al., 2020). Teachers had to think of new ways to monitor students' online learning and had to change assessment practice (OECD, 2020).

### 1.1 Formative assessment in COVID‐19 lockdowns

The forced online teaching period created new opportunities for assessment practices to evolve (Cahapay, 2020; Gikandi et al., 2011). For example, recent studies indicated that teachers used more formative assessment (FA) in their online teaching in the lockdown periods (Cahapay, 2020). FA is defined as a continuous process of gathering information about learning, to analyse and interpret this information and to take better decisions for further learning (Black & Wiliam, 2009). FA informed teachers what the online learning needs of students were and helped teachers to adapt their instruction and lesson plans to these needs. In other words, FA offered teachers opportunities to engage, guide and monitor students in their online learning (Chen et al., 2021). Another reason for teachers to use FA in online teaching was to overcome the difficulties they experienced with using summative assessments online. Teachers not only experienced reliability and validity issues with online summative tests (Gikandi et al., 2011), they also often found it questionable how to interpret summative outcomes, due to the uncertain times of trying out online lessons or learning in sometimes unsuitable home environments (Kinzie, 2020). The shifts made in assessment practice will probably change future (blended) assessment practices, with FA taking a more prominent place to monitor student learning (Cahapay, 2020). In the present study, the unique first COVID‐19 lockdown period allowed to study how Dutch secondary school teachers' use online FA practices. Following a previous study typifying teachers' FA practices in face‐to‐face education (Veugen et al., 2021), this study applies the FA cycle as a framework for describing teachers' online FA practices in depth, and in all phases of the FA process. This allows for comparing and finding opportunities of online, face‐to‐face or (future) blended forms of FA.

### 1.2 Teachers from the FA learning network

To assure that teachers do not lack assessment competence nor experience with FA, the present study focuses on a specific group of teachers from a FA learning network, who can be regarded as competent in applying FA in their face‐to‐face classroom (Veugen et al., 2021). In 2018, a FA learning network was established in the Netherlands, in which over 100 teachers from 13 secondary schools, teaching different subjects and grade levels, participated for a period of 4 years. Since the teachers of the learning network had the opportunity to implement FA practice in the classroom before, and they were forced to teach online during the first lockdown period, the question emerged how their gained knowledge and experience with FA helped them to design their online FA practice. In addition, due to their previous experience with FA, there was more chance that these teachers would succeed in adapting their FA practice online and thereby could reflect on what they experienced as differences between using FA online compared to using FA face‐to‐face. Furthermore, these teachers could further uncover the potential possibilities of online FA to boost future (blended) FA practice.

### 1.3 Literature review

In this literature review we first describe the FA process based on the FA cycle: A framework consisting of five aligned phases for teachers to implement FA in their (online) classroom. Then the challenges and opportunities teachers experienced with using FA online in previous studies are mentioned. Lastly the research questions are presented.

### 1.4 (Online) Formative assessment

In a recent study of König et al. (2020), which was conducted in the COVID‐19 lockdown period in Germany, many teachers did not succeed in implementing FA in online teaching. Other studies also indicated that teachers need to have the opportunity to develop new knowledge and skills about how to use FA online before they are able to implement the FA process (Anderson, 2008; Feldman & Capobianco, 2008). The sudden change to use FA in an online context could be difficult for many teachers. However, some teachers in these studies did succeed in implementing FA in their online teaching. More information is needed to uncover what successful teachers' FA practice looks like and how online FA could enrich face‐to‐face FA practice. FA is an interactive and dialogical process between teacher and student (Carless & Winstone, 2020). For teachers to successfully implement the FA process, that is for interactive and dialogical FA to take place and have an effect on teaching and learning, two conditions have to be met. The first condition is that all students should be actively engaged in the process, meaning that students are explicating their learning in some form. The second condition beholds that teachers and students should continuously monitor, evaluate and adapt their teaching and learning to meet the learning goals (Wiliam, 2006). These *pedagogies of engagement* and *contingency* are according to Wiliam (2006) necessary to move learning forward and therefore key to let FA succeed, also online.

### 1.5 Challenges and opportunities of online FA

Earlier research on how teachers implemented FA in an online context showed that teachers both experience new opportunities, but also identify challenges, to engage, guide and monitor student learning.

### 1.6 Opportunities and challenges to engage students online

Regarding the opportunities, studies indicated that teachers mainly use tools to let all students engage online in FA practice, for example via an online quiz or organizing (small) group discussions (Chen et al., 2021; Gikandi et al., 2011). Some studies revealed that student engagement was stimulated online due to diminishing constraints, such as time, peer and test pressure (Anderson, 2008; Filius et al., 2018; Gikandi et al., 2011). However, studies also reported some challenges for teachers in engaging all students in the online FA process, such as facilitating an interactive (feedback) dialogue with students about their learning (Filius et al., 2018; Van der Spoel et al., 2020). In these studies, teachers experience difficulties due to less handles to create small discussions, less student response and less synchronous interaction online. In other words, in online education there are new opportunities to keep students engaged thanks to online tools and methods. However, not all students are easily engaged behind a monitor and teachers experience difficulties to interact with students in, for example, lively dialogues online.

### 1.7 Opportunities and challenges in guiding and monitoring students

When looking into how teachers guide and monitor student learning, studies showed that gather responses from all students via online tools, create an overview via the used tools and provide more adequate and timely feedback (Dalby & Swan, 2019; Danniels et al., 2020; Gikandi et al., 2011). Some studies even showed that teachers give more individual feedback in the online classroom than in the face‐to‐face classroom (Basilaia & Kvavadze, 2020; Filius et al., 2018). Furthermore, it was found that teachers set up online environments for students to meet each other, so they could discuss their work and give each other peer feedback (Gikandi et al., 2011). However, Danniels et al. (2020) found that not all teachers analysed and used the gathered data to adjust their instruction and help students further. Filius et al. (2018) found that some teachers experience more difficulties in monitoring all students online, due to less interaction in an online lesson. Teachers often miss collecting information of student learning through students' non‐verbal communication and informal methods to ‘measure’ how students are doing in learning (Anderson & Rivera‐Vergas, 2020; Beebe et al., 2010). In conclusion, teachers are able to guide and monitor learning by using online tools that facilitate individual feedback. Still, the lack of interaction and non‐verbal communication makes online monitoring and guiding challenges as it does not allow teacher to ‘read’ how students are doing in their learning.

### 1.8 Added value of this study

However, these former studies all investigated the use of FA in hybrid forms of learning, where teachers used certain online tools to, for example, provide students with timely feedback, but often focused only on some phases of the FA process and did not study the alignment between different phases of the FA process (Gikandi et al., 2011; See et al., 2021). Moreover, simply using online tools does not automatically lead to FA practices that enhance student learning (Dalby & Swan, 2019). Not many studies until now have investigated FA practices as an ongoing process of aligned activities in online learning environments in secondary education (Cavanaugh et al., 2009; Chen et al., 2021; McLaughlin & Yan, 2017). More research is needed to investigate how teachers implement the FA activities of the FA process online and how they engage students in these processes to adapt teaching and learning (Danniels et al., 2020). Therefore, in this study we further explore if these challenges and opportunities also adhere when FA practice is quickly shifted to a complete online context and what other challenges and opportunities teachers experience.

### 1.9 The FA cycle

**FIGURE 1:**
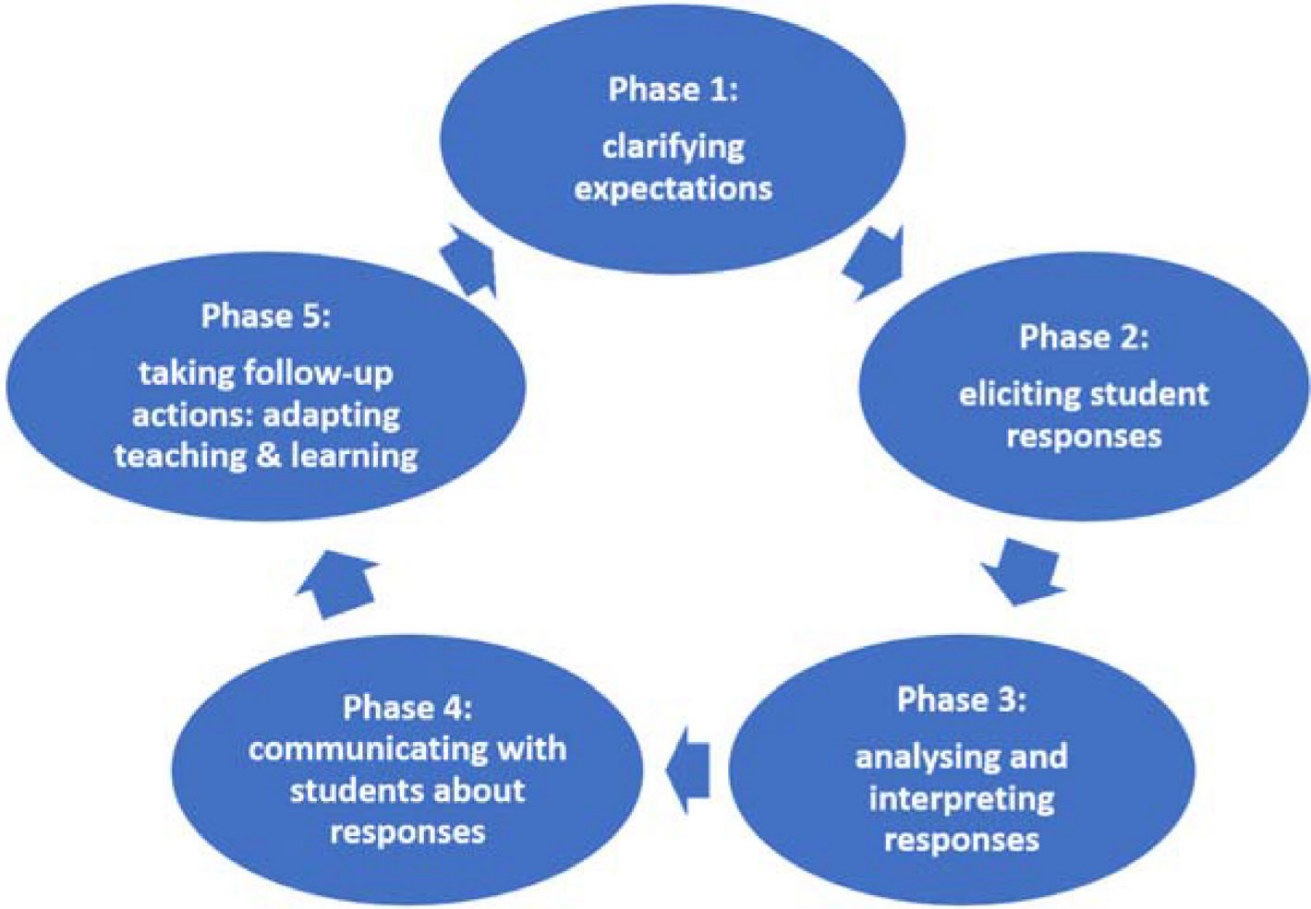
The FA cycle and the five phases (permission to use from Gulikers & Baartman, 2017)

Earlier research showed that the FA cycle is a useful and reliable model to operationalize the FA process and could help teachers to implement this process in their classroom (Gulikers & Baartman, 2017; Veugen et al., 2021). This model is also used in this study to disentangle and analyse teachers' online FA practices. The FA cycle describes concrete teacher activities within the FA process consisting of five aligned phases. Teachers should create opportunities for students to actively engage in FA within these five phases (Carless & Winstone, 2020). These five phases are: (1) clarifying expectations, (2) eliciting student responses, (3) analysing student responses, (4) communicating with students about responses and (5) taking follow‐up actions: adjusting teaching and learning (see Figure 1). Most of the time teachers start with clarifying their expectations (Phase 1) that include discussing the learning goals and success criteria with their students. Then teachers elicit student responses (Phase 2) in which teachers choose appropriate methods to gather information about student learning to reach the learning goals. The next step is analysing and interpreting these responses (Phase 3) to draw conclusions about where students are in their learning in relation to the learning goals. Teachers then communicate about these findings with their students (Phase 4), most of the time in a form of feedback. Lastly teachers think of follow‐up actions (Phase 5) in terms of adjusting their teaching or student learning activities or strategies. These decisions are on their analyses and feedback discussions with their students in the previous phases. If required, new or refined learning goals are set that allow for the next FA process to start.

Teachers participating in the present study developed their FA knowledge and skills based on the five phases of the FA cycle and collaboratively experimented with and reflected upon FA in their teaching in the learning network. Earlier research within this specific teacher group showed that these teachers were able to implement FA cycle activities of all phases in the classroom to some degree (Veugen et al., 2021). However, this study also showed that teachers found it most difficult to use activities to adjust teaching and learning to students' needs (Phase 5), to align the five phases in an ongoing process, and to use activities to activate students in the FA process in all phases. In the present study these face‐to‐face applied FA cycle activities of this specific teacher group will be compared with their implemented online FA activities.

### 1.10 Research questions

In sum, in this qualitative and exploratory study we will investigate how teachers of secondary education, who participated in a FA learning network, adapted FA cycle activities in their online education and how this differed from their FA activities in face‐to‐face education according to themselves, all in the context of the first COVID‐19 lockdown. We will further explore if and how these teachers used opportunities of online FA to engage, guide and monitor student learning and what challenges they experienced. Lastly, we will investigate what new developments in FA they experienced and what lessons these teachers learned from implementing online FA. This leads to the following research questions:

1. How do secondary school teachers, who are participating in a FA learning network, report to use the five phases of the FA cycle differently in their online teaching than in face‐to‐face education?
2. What challenges and opportunities in online FA do teachers experience to engage, guide and monitor students in their learning?
3. What FA developments and lessons learned do teachers report to have experienced due to the sudden transition to using FA online and how could these experiences help teachers to improve (future blended) FA practice?

## 2 METHOD

### 2.1 Design

This qualitative and exploratory study was conducted in the period of the first COVID‐19 lockdown between March and July 2020. We used a mixed methods design with a questionnaire, semi‐structured individual interviews and webinars with groups of teachers to gather data on the online FA practice of teachers. First the questionnaire was set‐up and spread among participants of the FA learning network. Second the interviews were planned with teachers who volunteered to participate, in which open questions were asked to gather information about teachers online FA practice. Third the results from the questionnaire and interview data were used as input for interactive webinars (also see Section 2.3). Webinars were organized to both meet teachers in their needs to develop knowledge and skills of online FA in collaboration with others in the learning network, as well as to collect additional data.

### 2.2 Participants

In total, 50 secondary school teachers of 17 different secondary schools in the Netherlands volunteered to participate in this study. However, not for all teachers data was collected with all three aforementioned methods: 17 teachers only completed the questionnaire, 30 teachers only joined a webinar and three teachers were only interviewed. Another three teachers completed the questionnaire and joined a webinar and three other teachers completed all three instruments (see Table 1).

**TABLE 1:** Division of the participants over the instruments (*n* = 50)

Instrument(s) used	Number of teachers
Questionnaire	11
Webinar	30
Interview	3
Questionnaire + webinar	3
Questionnaire + webinar + interview	3
Total	50

Due to the hectic short time period in which teachers suddenly had to switch to online education, not many teachers had time to take part in an interview, but most teachers did have an interest in being part of a webinar, which also helped them in designing and improving their online FA practice. Therefore we provided the teachers a choice in instruments, so if teachers would prefer to be part of a webinar instead of an interview they were able to do so. All instruments were designed to gather information about the online FA practice of teachers to answer the three research questions in different ways. The teachers of the whole learning network as well as the selection of these teachers who took part in this study represented a variety of subjects, education types and grade levels (see Table 2). Of the teachers who participated in this study were 20 male and 30 female. Since teachers started participating in the FA learning network from 2018, they were experienced with using FA in their practice for at least 1 year. This guaranteed that all teachers had at least basic knowledge and skills on, and experience with, FA. From the teachers who participated in the webinars, their name, subject and school were asked during the introduction of the webinar. From the other teachers, additional information was collected on the grade level(s) and type of secondary education they taught.

**TABLE 2:** Teacher characteristics

	Teachers	
	*n*	%
Gender		
Male		40
Female	30	60
Teaching upper or lower grades		
Lower (Grade 7,8,9)	6	18
Upper (Grade 10,11,12)	2	4
Both	14	28
Unknown	28	56
Subjects^a^		
Alpha	21	42
Beta	11	22
Gamma	13	26
Unknown	5	10
Education type^b^		
Vmbo‐bg	17	34
Vmbo‐t	0	0
Vmbo‐t and Havo	3	6
Havo	2	4
Havo and Vwo	9	18
Vwo	1	2
All types	5	10
Unknown	13	26
Joined FA learning network		
2018	10	20
2019	13	26
Unknown	27	54

^a^ Gamma, Geography, History, Economics, Societal Studies + also Environmental Studies, Art, Music, Gymnastics; Alpha, Dutch, English, German, French and Spanish; Beta, Physics, Mathematics, Biology, Chemistry and Technology.

^b^ The Dutch education system knows four education types: Practically oriented pre‐vocational education [*Dutch abbreviation: Vmbo‐bg*], theoretically oriented pre‐vocational education (*vmbo‐t*), senior general secondary education (*havo*) and pre‐university education [*vwo*].

### 2.3 Data collection

First, the questionnaire was constructed and made available in Qualtrics and spread by e‐mail to all teachers participating in the FA learning network. The questionnaire consisted of 17 open‐ended questions with no time nor word limitation and was developed by two authors in collaboration with the FA trainers facilitating the FA learning network to appropriately relate to the secondary school teachers. The 17 questions related to topics concerning the three research questions, such as how they used the five phases of the FA cycle and how this use differed from face‐to‐face implementation (research question one), what challenges and opportunities teachers experienced in engaging students and monitoring students' learning (research question two) and to what lessons learned and new developments using online FA led (research question three) (see Appendix A).Second, semi‐structured interview questions were created to gather more in‐depth information from the teachers to answer the three research questions. The interview questions were developed by the first two authors of this study, consisting of six questions (see Table 3) directly tapping into the three research questions

**TABLE 3:** Interview questions about online FA

Research question	Question
FA cycle practice (RQ1)	How do you use FA and the five phases of the FA‐cycle at this moment in online teaching?
FA cycle practice (RQ1)	How does your use of FA online differ from FA in face‐to‐face education?
FA cycle practice (RQ2)	How do you engage your students in this FA process?
FA cycle practice (RQ2)	How do you guide and monitor student progress at this moment?
Challenges and opportunities (RQ2)	What challenges/problems do you experience with online FA at this moment? And what chances/successes of online FA have you experienced until now?
Developments and lessons learned (RQ3)	What developments and lessons learned do you take with you from using online FA at this moment (for when you start teaching in the ‘normal’ classroom again)?

Third, five webinars of 90 min were scheduled in the months May, June and July. Teachers were asked to prepare their own FA examples using the five phases of the FA cycle, challenges, opportunities and lessons learned of doing online FA before the webinar started. During the webinars, the researchers and trainers of the FA learning network shared online FA examples, divided over the five phases of the FA cycle gained from the questionnaires and interviews, with the teachers. Together with the trainers and researchers, teachers discussed these examples and their own experiences of using online FA and compared these examples to their normal FA practice (to answer research question 1). The researchers and trainers also asked the teachers to share what challenges and opportunities they experienced using FA in the online context compared to face‐to‐face (to answer research question 2) and what developments they experienced and lessons learned they liked to keep into their FA practice for the long term (to answer research question 3). Teachers were sometimes given turns or took turns by themselves when they wanted to elaborate on an example or respond to another teacher. In the end all teachers shared some experiences and concrete examples of online FA activities in these group conversations, but not all teachers shared an equal amount of information. These group discussions were recorded and transcribed for analyses. The interviews and webinars were held via Microsoft Teams and recorded. All teachers were asked for consent before they were recorded.

### 2.4 Analyses

After the data collection, all the interviews and webinars were transcribed, anonymised, and analysed in Atlas‐ti.

### 2.5 Coding process

The first step in analysing the data of the three instruments was to create documents of the transcribed interviews and webinars, as well as one document containing all answers of the completed questionnaires, and to upload these in the Atlas.ti program.

As second step the first author created meaningful segments in all documents. To create a segment, data units were selected that touched upon topics related to one of the three research questions. These segments could vary from one sentence or a couple of words in the questionnaire data to long parts of texts describing a relevant topic in the interview and webinar data.

The third step was to code the segments of the total dataset. The codebook The following coding steps were drawn from Miles et al. (2020): first deductive, then inductive coding. Per code, the total number of segments and percentages of the total number of segments were calculated providing some quantitative indicators. Finally, analytic memos per code were created to make a narrative description per research question combining quantitative indications and qualitative analysis. Was finalized after several rounds of coding, discussing and correcting codes (see Appendix B). Codes were created that fitted the research questions for a first round of deductive coding (Miles et al., 2020). Related to the first research question, the five phases of the theoretical FA cycle were used as a codes (see Figure 1) and the code ‘alignment’ was added to grasp if and how teachers aligned the five FA phases. To answer the second research question, the initial codes directly connected to the research questions, resulting in the codes ‘opportunity of online FA’, ‘challenge of online FA’ and ‘FA different/similar’ were created. For the third research question two codes were created, namely ‘FA development’ and ‘tips/lessons learned’. In this first round of coding the first two authors both coded two webinars and two interviews and discussed the agreement of the codes and how to improve the coding process. During this first round of coding, new inductive codes relating to the second and third research questions emerged that were added to the first set of deductive codes (Miles et al., 2020). These included ‘student opportunity’, ‘student challenge’ and ‘students different’ relating to the second research question. For the third research question, added inductive codes were ‘support to develop FA’ and ‘prerequisite’. The first two authors both coded two webinars and one interview with this final version of the codebook to calculate the interrater reliability, resulting in a Krippendorffs' alpha of 0.76, which is acceptable (Krippendorff, 2018). The other documents were then coded with this final codebook by the first author. The fourth step was categorizing the segments of all documents per research question (see Appendix B). For research question one, for example, this resulted in segments of the questionnaire, interview and webinar data ordered in terms of the five FA phases and the code ‘alignment’. The number of segments per code and the percentages of the total number of segments were calculated and presented in tables in the results section.

The fifth step was to summarize the information per code. Therefore, analytic memos were created per code that described and summarized the information per code in a couple of sentences and resulted in a narrative description of the data for each research question (Miles et al., 2020) (e.g. see Table 4). By summarizing the information in this way, the dense information shared in an interview could be related to a short answer in a questionnaire. For example when a longer segment of an interview and a shorter answer on a questionnaire both described that they discussed learning goals with students in an online lesson to guide student learning (categorized in code ‘Phase 1’), they both were assigned to the analytic memo “The teachers discussed the learning goals with students online”. The number of analytic memos for each code were also added in the results as quantitative data.

**TABLE 4:** Examples of codes, analytic memos and segments per research question

Research question	Code example	Analytic memo example	Iconic example of segment assigned to the memo
1	Phase 1	Teachers discuss the learning goals with students in the online lesson.	‘When I start my lesson in the program LessonUp I first discuss what the learning goals are and what we are going to do’.
2	Student opportunity	Students are taking more ownership in their learning.	‘We noticed mostly… off course you hope for that… but the part of taking self‐ownership with students was something that was more visible. Autonomy, students can suddenly take control over their own learning. And students of whom you totally do not expect it are doing very well! Group pressure is gone, classroom distraction is gone and suddenly they start working!’
3	Support to develop FA	Teachers discuss together about how to use FA in online teaching.	Well, we are now evaluating with teachers what really works. And the first reaction of our managers, with whom we have had an interview, was that student learning has continued in a normal pace and that we are able to monitor the learning process of the students in different ways. And they used all different kinds of assignments and tools every week to do accomplish that’.

### 2.6 Calculating percentages

As sixth and last step we counted how many segments of *different* teachers were present in the analytic memos and converted these into percentages of the total teachers when described in the results section. Since some teachers repeatedly provided information about an analytic memo in a questionnaire, interview and/or webinar, these segments, describing similar information, were counted as one. For example, when a teacher shared that (s)he used learning goals to direct instruction in the online lesson in both the questionnaire, interview and/or during the webinar, these segments were counted as one teacher for that analytic memo. Then the percentages of different teachers who shared information per memo were calculated. The percentages do not mean that the other teachers did not use the activity mentioned in the memo, but that they just did not mention this activity in the questionnaire, interview or webinar. For example, when 68% of the teachers shared that they discussed learning goals and success criteria, this did not mean that the other 32% did not use this activity, just that they did not mention this activity in the questionnaire, interview or webinar. These percentages gave an idea of what analytic memos were more or less often mentioned by teachers. These analytic memos and their frequencies were used to qualitatively describe a narrative per code. These qualitative data were illustrated with iconic quotes found in the interview and webinar data. The iconic quotes were selected based on how well they clarified the data and therefore not depended on the teacher sharing the information. Teachers were given pseudo names in the analyses.

## 3 RESULTS

The results below are structured along the research questions. They first describe some quantitative results after which the analytic memos per code are qualitatively explained using iconic quotes of the teachers.

### 3.1 The online FA practice of teachers (Research question 1)

In total 723 segments were identified and coded within the questionnaire, interview and webinar data. Of these segments, 392 segments (54%) described online FA practices of teachers within at least one of the five phases. Onehundred and forty‐six of the 392 segments were coded with two or more phases and therefore were given the code ‘alignment’. Table 5 described which phases were mentioned together in one segment, insinuating that teachers aligned these phases to each other. In this table could be seen that Phase 1 is most often aligned with Phase 2 (18%) and that the alignment between the phases 2,3,4 and 5 showed the lowest alignment between phase 3 and 5 (22%) and the highest between Phases 2 and 3 (53%). In Table 6 the number of segments per code and number of created analytic memos could be found. The memos were summarized and illustrated with percentages of the total number of teachers and quotes of teachers.

**TABLE 5:** Alignment between the five phases in number of the total 146 segments

	Phase 1		Phase 2		Phase 3		Phase 4		Phase 5	
	Number of segments	%	Number of segments	%	Number of segments	%	Number of segments	%	Number of segments	%
Phase 1	–	–	–	–	–	–	–	–	–	–
Phase 2	26	18	–	–	–	–	–	–	–	–
Phase 3	18	12	53	36	–	–	–	–	–	–
Phase 4	15	10	46	32	50	34	–	–	–	–
Phase 5	10	7	32	22	43	29	49	34	–	–

**TABLE 6:** Number of segments and memos per code for research question 1

Codes of research question 1	Number of segments	% of total number of segments	Total analytic memos per code
Phase 1	134	19	21
Phase 2	175	24	18
Phase 3	99	14	14
Phase 4	128	18	16
Phase 5	101	14	10
Alignment	146	20	–

Regarding FA phase 1 (*clarifying expectations*), 68% of the teachers shared that they discussed the learning goals and success criteria with the students online. Teachers shared the learning goals and success criteria via assignments, powerpoints, digital tools or by discussing them during the lesson and used the learning goals and success criteria to shape their online lessons and to let students know what was expected of them in online learning. To make students more active during this phase, 24% of the teachers asked their students to discuss the success criteria based on examples of (previous) work before and during the online lesson:

We also shared some extra examples [of an assignment] before a Google Meet. And we let students categorize the examples. Which one do you think is really good and which one do you think is less good and why? That resulted in the success criteria that they used to describe a text, an e‐mail in this case (Barbara, Dutch teacher, havo/vwo, several grade levels).

Overall teachers mentioned that learning goals and success criteria became even more prominent in online teaching to guide teaching and learning than in face‐to‐face FA practice. Also students seemed to notice that they needed learning goals and success criteria more to guide their learning process, as one geography teacher, who taught mostly higher grades of vwo, noticed that students almost insisted on learning goals being provided during online lessons.

Regarding *eliciting student responses* (Phase 2), teachers had to find new ways to gather student responses that would fit the online situation. Most teachers (80%) shared that they used more online tools to collect data, such as LessonUp, Nearpod, online Exit tickets and Quizlet. Teachers (8%) remarked that, compared to face‐to‐face teaching, they had to think more consciously about what kind of student responses they wanted to collect and how they were going to collect these responses before the lesson. Teachers (16%) reported to collect individual student data more often via an online tool than in face‐to‐face education. Most teachers (64%) used more formal ways of gathering individual student data than in their face‐to‐face teaching. For example, teachers collected more responses via online student reports, student assignments or quizzes. During synchronous meeting time, asking questions remained an important informal strategy to collect student responses. Of the teachers, 44% reported using this strategy to identify and deepen their understanding of students' misconceptions, to uncover what was learned or to see how learning itself was going.

Regarding *analysing student responses* (Phase 3), 44% of the teachers shared that they focused more on individual results compared to face‐to‐face FA practice. Teachers (12%) reported that several online tools provided a clear overview that helped them to quickly analyse collected student data at an individual level. Similar to face‐to‐face situations, 36% of the teachers mostly searched for mistakes, misconceptions and gaps in understanding. Compared to face‐to‐face education, 36% of the teachers mentioned they more often let students analyse their own work, or the work of a peer, along the success criteria or a good example, before handing in their work. Of the teachers, 8% noted that students sometimes even analysed their own work without being asked to do so, which did not happen often in face‐to‐face situations.

When teachers *communicated with students about the results* (Phase 4), 54% of the teachers shared that they gave more individual feedback to students than in face‐to‐face teaching. Online teaching created more opportunities for many teachers to organize private feedback meetings with individual students or small groups. Reasons mentioned by teachers for this were that teachers and students had less instruction time online, creating more time for individual meetings, and that the online tools that provided a clear overview of the student progress in learning (Phase 3) helped teachers and students to provide timely and adequate (individual) feedback. Similar to face‐to‐face situations, 40% of the teachers shared that they provided feedback on what students had to improve in the assignment or task they made and less on the learning process itself. Only 4% of the teachers shared that they related their provided feedback to the learning goals addressed in Phase 1. Also, different than in face‐to‐face FA practice, 16% of the teachers mentioned that they often stimulated students to use an online feedback tool, checklist or rubric with the success criteria, to give feedback to themselves and to other peers.

With respect to the final phase 5, 64% of the teachers mentioned they *adjusted teaching and learning towards the students' needs* (Phase 5), of whom 14% mentioned that they did this more often and more planned than in face‐to‐face FA practice. Teachers frequently let students adjust their assignment based on their feedback, or let students make an additional assignment that fitted their needs in learning. In this way, teachers tried to differentiate to meet the students' needs in learning, and the online environment offered them new ideas on how to do this. Teachers (20%) even shared that they engaged students in discussions on possible follow‐up actions more, leading to some students thinking of a follow‐up action by themselves. The ease of organizing individual or small groups online meetings was experienced as facilitating these discussions, focussing on what the student still needed to learn and how the student was able to improve learning.

And for the students who, well, proved to have difficulties with these math assignments in their books, I thought of another type of follow up assignment. I asked them to videotape themselves explaining how to solve the math assignments, as if they were teaching. So I just turned it around. […] The students would use my feedback to first figure out the follow‐up assignment themselves and then videotaped their explanation of the assignment. So in that way I returned the assignment and could check if they did something with the feedback. (Emmet, physics teachers, vmbo‐t/ havo, all grades)

Although most teachers used the aforementioned practices, results also showed a variation in the extent to and the way in which teachers adopted online FA. Some teachers (8%), for example, found it more difficult to clarify learning goals online (Phase 1), due to less interaction with students online. One teacher, who taught French in all grades of havo and vwo and also was a team manager, mentioned that some colleagues did not create online opportunities for students to ask questions (Phase 2). Teachers (10%) shared that they only checked if student work was handed in online and did not analyse the work (Phase 3). Also, the quality of the feedback differed between teachers and some teachers (8%) shared that they did not know how to efficiently provide feedback online (Phase 4). Lastly, 8% of the teachers mentioned that they did not know how to adjust their teaching to the students' needs in the online situation (Phase 5). Two teachers who also had the role of team manager reported a wide variation in teachers' online FA practices within their school. One of these teachers (Gary, French, havo/vwo, all grades, and team manager) said that teachers who already used FA practices in face‐to‐face education adapted online FA activities more easily than teachers who had not used FA practice a lot before:

Well, it is very diverse. There were teachers who were doing very well, when you look at using FA online. For example Iroh, who made sure all the students knew what the learning goals were, who kept using feed‐up conversations with his students, in which students were asked to think about what aspects were important in learning. […] He used small assignments and quizzes in which he gave feedback on students' answers. He teaches at a high level, I think. And on the other hand I saw a teacher who only gave students an assignment with a deadline, as in ‘then you have to finish the assignment, good luck’. Who did not even answered questions students e‐mailed to him.

### 3.2 Opportunities and challenges of online FA for engaging, monitoring and guiding student learning (Research question 2)

Of the total 723 segments, 325 segments (45%) were coded with one of the six codes to answer research question two. The number of segments per code was described in Table 7. Also noted in this table is the number of memos in which the segments were categorized to describe the dataset. Similar to the results section of research question one, the memos were described in this section, percentages of the teachers who shared information regarding the memos were included and some quotations were added to illustrate the shared information.

**TABLE 7:** Number of segments and memos per code for Research question 2

Codes of Research question 2	Total number of segments	% of the total number of segments	Total analytic memos per code
Challenge of online FA	89	12	19
Opportunity of online FA	74	10	12
FA different/similar	19	3	7
Student opportunity	53	7	11
Student challenge	20	3	9
Student different/similar	70	10	12

### 3.3 Opportunities and challenges in engaging students

Teachers used many opportunities of online FA to stimulate students' engagement in the FA process, for example by initiating more self‐ and peer assessments. Teachers let students formulate the success criteria (Phase 1), stimulated students to ask questions also by integrating online tools in their lessons (Phase 2), let students analyse their own and their peers' work (Phase 3), let students give feedback to themselves and their peers (Phase 4) and let students think of a follow‐up action to improve their learning (Phase 5) (see also results at RQ1). Of the teachers who were able to use these FA practices online, 24% reported that students used the opportunities online FA offered them and noticed that students were more engaged during the online lessons, asked more questions and took feedback more seriously:

They got feedback per subject every week on reaching the learning goals in the form of a ‘Good’, ‘OK’ or ‘Not Sufficient’ evaluation, and that stimulated them enormously to work for the ‘Good’ evaluation. So they really used the feedback to improve their learning and came to teachers to ask things like ‘I have an OK at this moment, what do I need to do to get a “Good”?’ And normally we got those kind of questions way less during lessons. (Barbara, Dutch, havo/vwo, all grade levels).

Regarding shared opportunities for students of online FA, three teachers reported that most students were quite able to provide useful feedback to themselves and that the success criteria helped them to evaluate the quality of their own work. These teachers experienced that the students took more responsibilities in regulating their own learning compared to when they used FA practice face‐to‐face. Furthermore, 22% of the teachers mentioned that students asked more questions than in a face‐to‐face lesson. A reason for this, according to some teachers, was that the online situation made it less easy to ask questions to their peers and students had to learn more on their own than in face‐to‐face education. Two teachers even noticed that students started to work together and asked each other for help, without the teachers stimulating this. Besides the created opportunities for students to be more involved in the FA process, 14% of the teachers reported that the lack of group pressure, summative test pressure and time pressure stimulated students to be more engaged in the FA process.

However, 10% of the teachers reported that it was sometimes difficult to engage all students online. These teachers shared that since the lockdown period asked them to quickly shift their education, they felt that they needed to guide their students more in these uncertain times and thereby experienced challenges in engaging students in the FA process. They used more teacher‐centred activities and set goals for students (Phase 1), monitored student learning (Phases 2 and 3) and directed the students in what was needed to improve (Phases 4 and 5) (also see results of RQ1), but had difficulties in using more student‐centred activities, such as stimulating self‐ and peer assessment online. This resulted in students experiencing challenges of online FA, such as less motivation to actively participate in the online FA process or less student responsibility in regulating their own learning. According to 22% of the teachers, these problems occurred mostly with students who already had difficulties in stimulating themselves to be actively engaged and motivated in learning and preferred learning with their peers, which happened less online.

And that was…, it was very different in how active you were and how active your students were during the lesson. That I as a teacher was maybe more active than the students. I mean, they did their work fine, but normally they are actively increasing their knowledge and I am there to support them. And I noticed that this was the other way around during the online period. That maybe is just the way it was, but still it was a bit of a pity. (Dory, English, havo/vwo, all grade levels)

### 3.4 Opportunities and challenges in monitoring and guiding students

Teachers saw new opportunities to guide and monitor student learning by using FA online. As a start, 16% of the teachers reported to have more time for the FA process, since their online lessons were shorter and there was less distraction during the lessons by, for example, interrupting students. This gave teachers the opportunity to make more time for guiding and monitoring, especially, individual students. Teachers (10%) experienced it to be easier to make time available during lessons (e.g. via BreakOut Rooms) and after online lessons to answer these questions individually or in small groups in online meetings. In addition, 6% of the teachers shared that the use of online tools helped them to collect student data of all students and to provide an overview of where students were in their learning (also see Phases 2 and 3 at RQ1). Overall 24% of the teachers noted that online FA created more opportunities to individually help students with their questions and to provide them with (individual) feedback, compared to face‐to‐face education, as was earlier described in phase 4 (RQ 1).

I noticed they just kept handing it (work) in. So they liked it to keep getting feedback and I also said ‘Look, normally I would walk around and look at your work for a couple of minutes to provide feedback. But I also want you to keep improving yourself at this moment. That you could see what goes well already. That you are prepared for next year’. And well yes, they really kept on turning in work and that is different compared to groups in which I do this (FA) less, like the upper classes for example (Dory, English, havo/vwo, all grade levels).

However, teachers also experienced challenges to guide and monitor students online. Many of the teachers (44%) shared that the lack of interaction in the online FA process was a main challenge. This lack of interaction resulted in teachers finding it difficult to keep the interactive ‘on the fly’ ways of FA in the online setting, such as reading non‐verbal communication to see where learning was going. The online context also made it more difficult, as 20% of the teachers reported, to directly help students during learning: “There was a delay in giving feedback, because they sent me the pictures, I provided them feedback and then a week had already passed. So the delay hindered a lot.” (Finn, arts, vmbo‐bg, all grade levels). Three teachers noted that some students were difficult to monitor in their learning, since they did not hand in their work nor ask questions. Therefore, the teachers found it difficult to figure out the problems and misconceptions these students had in learning. Teachers mentioned they had to think of new ways to provide online feedback to overcome these challenges, such as letting students provide feedback to themselves or their peers online.

### 3.5 Developments and lessons learned to improve FA (Research question 3)

To answer Research question 3, 261 segments were coded with one of the four codes related to this question. In Table 8 the number of segments per code and created analytic memos could be found. The memos were summarized and illustrated with percentages of the total number of teachers and quotes of teachers.

**TABLE 8:** Number of segments and memos per code for Research question 3

Codes of Research question 3	Total number of segments	% of the total number of segments	Total analytic memos per code
FA development	78	11	11
Support to develop FA	60	8	12
Prerequisite	27	4	9
Lessons learned/tips	96	13	19

### 3.6 Developments in (online) FA practice

Regarding developments in FA practice, teachers reported that the lockdown challenged them to some fundamental discussions on, and developments in, assessment. Teachers (26%) shared that they started to rethink the curriculum and what the essentials of their subjects were when they had to shift their FA practice to online. These teachers mostly used curriculum goals or earlier developed learning goals to establish their new lesson plans or educational programs. Some teachers (10%) even started to (re)formulate learning goals and success criteria to guide the online teaching and learning:

Well it is funny. I had a first grade and because we were working in periods, we kept the same lesson content, only we had to cover it in two times 30 min instead of 50. And then I discovered that I came more easily to the core of the subject. I reconsidered the learning goals, got rid of half of them and, to my own astonishment, noticed that the lesson was better than before! It really forced me to come to the core elements of my subject! (Luna, geography, levels of all types of education, lower grades).

As for the *support* teachers shared they received to adapt FA practice, 16% of the teachers mentioned that they collaborated with colleagues to reformulate learning goals for their lessons and to think about the essential knowledge and skills students needed to know. These conversations sometimes even led to rethinking educational visions, what good learning beholds and how assessment systems at school should operate, as illustrated by Barbara (Dutch, havo/vwo, all grade levels): ‘It (the online teaching period) forced us enormously to reconsider everything in teaching and to think OK, what do we find really important? And how are the students going to learn? And where do they need guidance and where not?’ All these questions are vital for assessment, summative as well as formative.

The way of assessing also developed due to new ideas of what teaching and learning should be about. Summative assessments were often not used during the online teaching period, because of difficulties in not being able to check if students looked things up in their book or got help from a parent or peer. Therefore, teachers thought of more formative ways of assessment to monitor their students. Compared to face‐to‐face education, 10% of the teachers shared that FA became more important in online education for the purpose of keeping an eye on students' learning progress. This made 8% of the teachers realize that they had to think more explicitly about what data they wanted to gather and analyse to make better decisions on whether students reached the learning goals or not and what to do next. In other words, the online situation made teachers plan their FA process more beforehand, making them design and implement activities of the five phases in their online FA practice. This is illustrated by Dory, an English teacher who taught all grades of havo and vwo:

So what I do, is to think really well of what I want to measure. What do I really want to know? What is my priority? And that is what I am measuring. And that is what I also let the students check for themselves first before handing in their work, so they also know what I expect of them.

Teachers also shared that for these discussions about the essentials in teaching and assessment to take place between colleagues and to enhance collaboration, some *prerequisites* were needed, such as time, access to digital learning environments and tools, and management support to, for example, facilitate workgroups.

### 3.7 Lessons learned to improve future (blended) FA

The lessons learned teachers intended to keep included certain FA practices of online FA that improved their FA practice. For example, 26% of the teachers experienced the added value of more time for individual feedback. They intended to keep enough individual time for students to ask questions (online), to provide them with individual feedback and to more explicitly help them to move their learning forward, so students would be stimulated to take student ownership in learning.

Well for me the biggest lesson learned was the importance, and this had been said before, of individual feedback or discussing things in very small groups, so two or three students. And taking time for that. I really saw, and that was an advantage of this period, that it was possible to have one on one contact in tranquillity with students. And we have to find a way to also create time for this in the normal lessons (Newt, science, havo/vwo, all grades).

Teachers (16%) mentioned that they wanted to keep using the new online tools to collect student responses in their lessons (as they used in Phase 2 in online FA, also see RQ1). Two teachers shared that they thought of keeping the online classroom environment to use for individual feedback conversations and student questions, since they experienced that some students felt more at ease to talk freely in these private online meetings.

One school already reported on very concrete ideas on how to change their FA practices after ‘going back to normal’: They wanted to integrate the online FA process with the face‐to‐face FA process in their near teaching. During the lockdown period, teachers of this school evaluated students' learning weekly along the learning goals for all subjects. Students could see their evaluation in an online system and got the chance to improve their work the week after. They could ask questions and got feedback from the teacher. Students shared that they appreciated this formative way of learning and liked it better than the previous face‐to‐face situation in which much more summative grading was used. Students argued that this FA practice informed them where they were in learning and gave them the possibility to improve themselves every week. Therefore, teachers of this school wanted to keep this system active when they could teach face‐to‐face again and made plans to integrate online FA with face‐to‐face FA practices in their education. They explicitly used the FA cycle to think about how to do this: They planned to use the online context to share the learning goals (Phase 1), let students make and hand in assignments (Phase 2) so teachers could already analyse them (Phase 3). In individual online meetings, or meetings face‐to‐face at school, teachers and students could provide feedback to each other (Phase 4) and already think of and use follow‐up actions (Phase 5).

## 4 DISCUSSION

This study leads to several points for discussion that allow for deducing lessons learned to improve FA in educational practice. Also some limitations and directions for future research will be discussed in this section.

### 4.1 Teachers' online FA practice compared to face‐to‐face FA

This study shows that teachers, who had former knowledge of, and experience with using FA practice in the classroom, are able to adapt the activities of the five phases of the FA cycle (Gulikers & Baartman, 2017) to their online teaching to engage, guide and monitor their students. Results also indicate that teachers are able to align FA practices of different phases. Earlier research on online FA mostly showed that teachers used *parts* of the FA process, for example by the use of online tools to collect student responses (Phase 2) (Gikandi et al., 2011; See et al., 2021). This study demonstrates that teachers use *all phases* of the FA process in their online teaching, even adjusting teaching and learning (Phase 5) which is mostly found most difficult to implement (Heritage et al., 2009; Veugen et al., 2021; Wylie & Lyon, 2015). One of the reasons for this could be that these specific teachers involved in this study were already used to implement FA strategies of the five phases in their face‐to‐face FA practice. In the study of König et al. (2020) it was also argued that the assessment competence of teachers allowed teachers to adapt their FA practice to the forced online context. Second, FA takes a more prominent place in monitoring student learning in this forced online teaching period, as summative assessments are less used due to for example validity and reliability issues (Cahapay, 2020). Teachers had to plan their FA practice more in this sudden online situation in which they could rely less on the regular summative assessments. Teachers are also less able to use and rely on ‘on the fly’ FA which they mostly used in face‐to‐face situations (e.g. walking around in the classroom to see how learning was going) (Gikandi et al., 2011). This context requires them to think more explicitly of what data they want to collect and why with FA, leading to more *decision driven data collecting* (Wiliam, 2014a). In doing so, teachers make the FA process more learning goal directed, more complete and ongoing than in their face‐to‐face teaching. These are important lessons for improving FA practice in online as well face‐to‐face teaching. The sudden online situation made schools choose to relief many summative assessment and turn to more FA. This situation required teachers to put more effort in planning FA, as part of their educational design (Boud & Molloy, 2013). This seemed to pay off in the current study, leading teachers to more learning goal oriented and complete FA cycle processes, including the final—crucial—Phase 5 of actually adjusting teaching and learning to close that gap in students' learning. The study also shows the importance of professional development programs to help teachers develop FA skills and practices. All teachers in this study had previous knowledge and experience of how to implement FA in their face‐to‐face practice, which seemed to help them also use FA in the sudden online teaching situation.

### 4.2 Using opportunities of online FA

The unique context of this COVID‐study creates new opportunities for teachers in using FA online. Teachers experience and use new opportunities in online FA to engage, guide and monitor students compared to their former face‐to‐face FA practice, which fosters both a *pedagogy of engagement* and *contingency* (Wiliam, 2006). Regarding stimulating a pedagogy of engagement, teachers search for new ways of engaging students in FA, since the online learning process was more individualistic and less interactive than in face‐to‐face classroom practice (Anderson, 2008; Filius et al., 2018). Online tools help teachers to engage all students in active participation (Chen et al., 2021; Gikandi et al., 2011). As to stimulating contingency, online tools combined with more time due to shorter lessons online, help teachers to create an overview of all students' progress. This, in turn, helps them to formulate more individual feedback (Phase 4) and to think of follow‐up actions for each student (Phase 5). This finding underlines earlier research that found that teachers are able to provide more individual feedback in online FA (Basilaia & Kvavadze, 2020; Filius et al., 2018). In addition, not only do teachers themselves provide more individual feedback, they also stimulate more self and peer assessment practices to allow student to monitor and guide their own learning in this online context. Students are stimulated to compare their own work to that of a peer or to the success criteria which leads to more internal feedback and helps students to improve their own work (Nicol, 2020). Teachers, who are able to use these online FA practices experience that their students ask more questions and ask for more feedback (cf. Filius et al., 2018). This provides an interesting opportunity of using FA online, since earlier studies found that in face‐to‐face FA teachers had most difficulties with initiating these self‐ and peer assessment activities (e.g. Hawe & Parr, 2014; Volante & Beckett, 2011). Another opportunity teachers experience of using FA online is that teachers note that less group, time and test pressure also stimulates students to actively take part in the online FA process (Anderson, 2008; Gikandi et al., 2011). This might be an interesting result for teachers, policy makers and others to further uncover how face‐to‐face education could overcome these issues related to pressure that apparently are less problematic in online learning. Thus, the study confirms earlier findings on possibilities on online FA. Especially in engaging all students in the FA process, incorporating more self‐ and peer assessment activities and using online tools for analysing student results and providing more individual feedback. If teachers are able to more sustainably implement these practices and in combination with face‐to‐face practices, FA practice can be further optimized and foster more student engagement, ownership and learning (Wiliam, 2006).

### 4.3 The main challenges in online FA

This study also indicates that teachers need to design opportunities for students to engage in the FA process for positive student effects to take place, such as students taking ownership in their learning (Black & Wiliam, 2009; Carless & Winstone, 2020). Especially teachers who share that they find it difficult to engage their students in the online FA process seem to fall back on more teacher‐centred FA practice, that leads to students taking less ownership in their learning and less student motivation for learning. An explanation for this finding might be that these teachers already found it difficult to stimulate self‐ and peer assessment in face‐to‐face education and feel less competent in using these FA strategies (Wylie & Lyon, 2015). Another reason could be that teachers find the sudden shift to online difficult to make and thereby find it difficult to give students more responsibility in the FA process (Hartshorne et al., 2020; Van der Spoel et al., 2020; Wylie & Lyon, 2015). Finally, in line with a study of Beebe et al. (2010), one of the main challenges for teachers in online FA is to directly help students with their difficulties and to figure out what misconceptions and problems students have in their learning. Similar to earlier studies (Anderson & Rivera‐Vergas, 2020; Filius et al., 2018; Van der Spoel et al., 2020), teachers in this study often miss the interaction with students online and reading non‐verbal communication as a way to see how learning is going. In other words, the use of FA practice ‘on the fly’ in online teaching remains a challenge that slows the informal FA process. This challenges teachers to find new ways to keep students engaged in the online FA process and to guide and monitor student learning. Thus, while several teachers found many opportunities in online FA—e.g. for active engagement, more self, peer and individual assessment—other teachers found it harder to transfer part of this responsibility to students. Stimulating and organizing active collaboration and sharing among teachers would allow for more support and inspiration in this respect.

### 4.4 Fuelling the assessment discussion in schools

In addition to the previous studies (Cahapay, 2020; Gikandi et al., 2011), this study shows interesting additional new developments in response to online FA experiences leading to new assessment perspectives and discussions within schools. Teachers experience how FA could help them to evaluate students' learning progress, without depending primarily on grades and formal summative tests. Some teacher teams even start to reform their assessment programs, in which they plan on using less summative assessment and more FA. This study brings to light that the forced COVID‐19 situation creates opportunities for teachers and schools to rethink their vision on education and assessment. This could lead to more balanced assessment programs in education, in which FAs and summative assessments could be more aligned with a focus on learning (Cairns, 2020; Tormey et al., 2020; Wiliam, 2014b). Teachers also intend to make use of their lessons learned and opportunities of online FA in their future blended FA practice, by combining opportunities of online FA with advantages of face‐to‐face FA to improve FA practice. As one school example in our results on Research question 3 shows, online FA could be used to provide more planned and complete FA processes by engaging, guiding and monitoring student learning with the use of online tools and providing more individual feedback, while in face‐to‐face education teachers could use the more ‘on the fly’ forms of FA to directly help their students with their questions and stimulate more interaction and feedback dialogue to determine where students are in their learning and what to do next. Furthermore, many teachers of this study experience that they are able to design and use all five phases of the FA cycle and align them with each other, which is already an improvement in their FA practice (Veugen et al., 2021).

To conclude, the unique COVID‐19 context made teachers rethink their teaching and assessment practices and helped them to also think more critically in why and what to assess. In some schools this even fuelled a discussion on their educational and assessment vision, discussing the balance between summative and FA, as well as on a future blended FA practice.

### 4.5 Limitations and future research

This study investigates teachers' online FA practice in the COVID‐19 lockdown period. Some limitations of this study need to be mentioned to interpret its conclusions. First, this study does not represent the population of secondary school teachers in the Netherlands. We purposefully had chosen teachers who were already experienced in applying FA in their practice, since we expected these teachers to be able to adapt their FA practice to online and use their former knowledge and skills of FA to implement interesting FA online practices. Therefore, the results found in this study may differ from those in another less experienced teacher group. Also, of the teachers participating in the learning network only those that volunteered and were motivated to participate in this study were represented. However, this population of teachers does teach us important lessons on the opportunities of online FA. Second, the study was conducted in a rather unique context of a lockdown period, in which teachers were forced to teach online and needed to change their education rapidly. Teachers did not have much time and sometimes lacked support or opportunities to learn new ways, but had to improvise mostly to keep education running. Therefore, comparisons with other studies of FA practices in online contexts have to be made carefully and could, for example, show different FA practices if teachers have more time to adapt, such as more technological competence (Van der Spoel et al., 2020). Third, this study was set up as quickly as possible to collect data of teachers' FA practices during the period of the first lockdown, which made it difficult to pilot test the instruments. However, we did try to meet this limitation by using a mixed method design to compare the data of three instruments with each other to further verify and deepen the answers given in relation to the three research questions. Additionally, since this study was an explorative study we used open questions in all instruments to gather the experiences of teachers without directing their thoughts to certain closed topics or opinions. Therefore, as mentioned earlier in the results, the percentages noted in the results section have to be interpreted carefully. Fourth, relating to the short time span of this study and its aims, we did not collect much background information of the teachers, such as prior digital skills or teaching experience. We acknowledge that this information could be valuable to interpret the results on an even deeper level. However, we do know that teachers all had previous experiences with using the FA cycle practices in their teaching, therefore making the teachers more unified than different from each other in this respect.

This exploratory study shows that online FA has potential to improve FA practice, in terms of engegement and responsiveness (Wiliam, 2018), and especially in combination with the advantages face‐to‐face FA could offer. More in‐depth research is needed to investigate the impact of online FA on improving teaching practice and student learning, engagement and ownership. Also findings what a good balance between online and offline FA could be needs further study. Another interesting scope for future research would be to investigate from a student perspective how they experience to engage in online and blended forms of FA practice and to what differences this leads in students taking ownership in their learning. Lastly, future research has to show if and how teachers keep their intended learned lessons and improve their future (blended) FA practice.

### PEER REVIEW

The peer review history for this article is available at https://publons.com/publon/10.1111/jcal.12699.

## Data Availability

The data that support the findings of this study are available from the corresponding author upon reasonable request.

## APPENDIX A QUESTIONNAIRE ABOUT ONLINE FA

**Table:**

Number	Question
1	What did you do to come to the core of your subject/to decide what lesson content you were going to cover in the online teaching period? And how did you collaborated in making these decisions with your colleagues?
2	How do you monitor at this moment how your students are reaching the intended knowledge and skills? What tools/support do you use to achieve this?
3	In which ways do you monitor how students are regulating their learning (at home)?
4	Are you able to see how students differ at this moment? If so, what differences do you see in students (ways of) learning?
5	Phase 1: How do you clarify your expectations at this moment? Do you make use of examples?
6	Phase 1: Is this different compared to how you used to do this in face‐to‐face education? And if so, how?
7	Phase 2: In which ways do you gather student responses at this moment? What kind of assignments, activities or tools do you use?
8	Phase 2: Is this different compared to how you used to do this in face‐to‐face education? And if so, how?
9	Phase 3: In which ways do you analyse and interpret the student responses at this moment? And what role do you give the student in this process?
10	Phase 3: Is this different compared to how you used to do this in face‐to‐face education? And if so, how?
11	Phase 4: In what way and when do you communicate with students about responses at this moment?
12	Phase 4: Is this different compared to how you used to do this in face‐to‐face education? And if so, how?
13	Phase 5: How do you help students in taking a follow‐up action in their learning at this moment? Can you give an example of how you adjust your teaching to the needs of students?
14	Phase 5: Is this different compared to how you used to do this in face‐to‐face education? And if so, how?
15	Do you learn together with colleagues about how to use monitor online student learning? And if so, could you give an example of your collaboration?
16	In what way does your school leader guide/support you in how you could monitor student development online? (For example, are there guidelines, agreements, tools available?)
17	Do you have an inspiring example of yourself, or from a blog or online tool, about how you could use FA online?

## APPENDIX B CODEBOOK AND THEMES OF ONLINE FORMATIVE ASSESSMENT

**Table:**

		Codes	Research question
Code number	Code name	Description	
1	Phase 1	Activity/activities of phase 1	1
2	Phase 2	Activity/activities of phase 2	1
3	Phase 3	Activity/activities of phase 3	1
4	Phase 4	Activity/activities of phase 4	1
5	Phase 5	Activity/activities of phase 5	1
6	Alignment	The teacher describes alignment between two or more phases	1
7	Challenge of online FA	Something mentioned by the teacher about online FA that is a challenge to engage, guide and monitor student learning	2
8	Opportunity of online FA	Something mentioned by the teacher about online FA that is an opportunity to engage, guide and monitor student learning	2
9	FA different/similar	The teacher shared that online FA is different from or similar to face‐to‐face FA, but does not distinct if this is a challenge or opportunity to engage, guide and monitor student learning	
10	Student opportunity	The teacher noticed students using the opportunities of online FA to engage, guide and monitor their own learning	2
11	Student challenge	The teacher noticed students struggling with the challenges of online FA to engage, guide and monitor their own learning	2
12	Student different/similar	The teacher noticed that students responded similar/differently in engaging, guiding and monitoring their own learning	2
13	FA development	The teacher mentioned something he/she has learned to adjust FA practice, change his/her knowledge or stimulate his/her own development in FA	3
14	Support to develop FA	The teacher mentioned collaborating with colleagues to develop online FA or gathering knowledge from (a) resource(s) to inform his/her own online FA practice	3
15	Prerequisite	The teacher mentioned what is necessary in online teaching to be able to use online FA	3
16	Lessons learned/tips	The teacher mentioned something that he/she finds (not) valuable of online FA practice and has come to the insight due to the online teaching period	3
